# Using Gelatin Nanoparticle Mediated Intranasal Delivery of Neuropeptide Substance P to Enhance Neuro-Recovery in Hemiparkinsonian Rats

**DOI:** 10.1371/journal.pone.0148848

**Published:** 2016-02-19

**Authors:** Ying-Zheng Zhao, Rong-Rong Jin, Wei Yang, Qi Xiang, Wen-Ze Yu, Qian Lin, Fu-Rong Tian, Kai-Li Mao, Chuan-Zhu Lv, Yi-Xiáng J. Wáng, Cui-Tao Lu

**Affiliations:** 1 College of Pharmaceutical Sciences, Wenzhou Medical University, Wenzhou, Zhejiang 325035, China; 2 The Second Affiliated Hospital of Wenzhou Medical University, Wenzhou, Zhejiang 325000, China; 3 Hainan Medical College, Haikou, Hainan 570102, China; 4 Taizhou Traditional Chinese Medical Hospital, Taizhou, Zhejiang 318000, China; 5 Biopharmaceutical R&D Center of Jinan University, Guangzhou, Guangdong 510000, China; 6 Department of Imaging and Interventional Radiology, Faculty of Medicine, The Chinese University of Hong Kong, Prince of Wales Hospital, Shatin, New Territories, Hong Kong SA; University of Helsinki, FINLAND

## Abstract

**Purpose:**

Intranasal administration of phospholipid-based gelatin nanoparticles (GNP) was prepared to investigate the neuro-recovery effects of neuropeptide Substance P (SP) on hemiparkinsonian rats.

**Methods:**

The SP-loaded gelatin nanoparticles (SP-GNP) were prepared by a water-in-water emulsion method and possessed high stability, encapsulating efficiency and loading capacity. PC-12 cells were used to examine the growth enhancement of SP-GNP *in vitro* by MTT assays and flow cytometry (FCM). The therapeutic effects of SP-GNP on 6-hydroxydopamine (6-OHDA) induced hemiparkinsonian rats were assessed by quantifying rotational behavior and the levels of tyrosine hydroxylase (TH), phosphorylated c-Jun protein (p-c-Jun) and Caspase-3 (Cas-3) expressed in substantia nigra (SN) region of hemiparkinsonian rats.

**Results:**

PC-12 cells under SP-GNP treatment showed better cell viability and lower degree of apoptosis than those under SP solution treatment. Hemiparkinsonian rats under intranasal SP-GNP administration demonstrated better behavioral improvement, higher level of TH in SN along with much lower extent of p-c-Jun and Cas-3 than those under intranasal SP solution administration and intravenous SP-GNP administration.

**Conclusions:**

With the advantages of GNP and nose-to-brain pathway, SP can be effectively delivered into the damaged SN region and exhibit its neuro-recovery function through the inhibition on JNK pathway and dopaminergic neuron apoptosis.

## Introduction

Parkinson’s disease (PD) is a chronic disorder of the central nervous system (CNS). PD is caused primarily by progressive loss of dopaminergic cells in the substantia nigra (SN) region, which leads to bradykinesia, muscular rigidity, resting tremor, and postural instability[1, 2]. PD affects 1%-2% of the population above the age of 60 [3]. Despite the progress for therapy of Parkinson’s disease, it remains a difficult disease for clinical management.

Neuropeptide Substance P (SP) is a member of the tachykinin peptide family that is involved in the regulation of many biological processes. SP is a major mediator of neuroimmunomodulatory activities and neurogenic inflammation within the central and peripheral nervous system [4]. SP-containing neurons are widely distributed throughout the central and peripheral nervous systems, especially in the SN region [5]. From *in vitro* experiments, SP can protect dopamine (DA) neurons from neurotoxicity, decrease neuron apoptosis and enhance cell growth [6]. From i*n vivo* studies, the expression of SP as well as DA is significantly reduced in the substantia nigra (SNc) of hemiparkinsonian rats and PD patients, which results in increased DA deaths and limited expression of tyrosine hydroxylase (TH) [7]. Researchs have also shown that SP plays an important regulatory role on dopaminergic pathways, particularly the nigrostriatal pathway [8]. After SP or SP receptor antagonist treatment for the rat model of Parkinson's disease, the content of striatal dopamine and its metabolites increase, and PD symptoms improve [9, 10]. In rats with substantia nigra partially damaged by 6-0HDA, systemic administration of SP before and after the damage promotes functional recovery of Parkinson's disease [10, 11].

Abnormally activated c-Jun N-terminalkinase (JNK) pathway is one of the important mechanisms leading to DA neuronal apoptosis in SN region [12]. JNK is one of the important members of mitogen-activated protein kinase, MAPK family. It has an important regulatory role for a variety of cells including nerve cells for their growth, differentiation, survival, and apoptosis [13]. Studies have shown that abnormal activation of JNK signaling pathway can activate downstream signaling pathways, leading to the death of DA neurons. Thus JNK signaling pathway plays an important role in the dopaminergic neuron apoptosis in Parkinson's disease [14]. By inhibiting the abnormal activation of JNK pathway, SP can execute certain therapeutic effects for Parkinson’s disease [15, 16].

Dopaminergic neuron apoptosis is also one of major causes of Parkinson’s disease [17]. The apoptotic process mainly results from protease cascade process mediated by Caspase family member. Caspase-3 has a vital role in the reaction process. From previous studies, 6-OHDA can induce apoptosis in PC12 cells by activating caspase and pro-apoptotic factor as well as transduction of Bax factor [18]. 6-OHDA injection into the rat brain can be used to induce the apoptosis of DA neurons in substantia nigra [19]. Researchs have also found that SP can effectively regulate the expression of caspase family proteins and cell apoptosis, thereby inhibit the symptoms of many diseases including cancer [20], spinal cord injury [21] and Parkinson’s disease [16]. Meanwhile, JNK signaling pathway downstream mitochondrial malfunction can lead to over-expression of Caspase-3 protein, ultimately leading to apoptosis or degenerative death of cells [13].

SP can play an important role in the recovery of diseased dopaminergic neuron through the deactivation of JNK pathway and decreasing neuron apoptosis. However, as a macromolecule neurokinin, it is not likely that SP administrated orally or intravenously could cross the blood-brain barrier (BBB) and its role in the treatment of brain diseases is therefore significantly restrained.

Intranasal delivery of biologics has been proved as a noninvasive and efficient strategy [22]. From previous studies, drugs or particles with a size below 300 nm could bypass BBB and play therapeutic roles at certain regions inside the brain [23, 24]. With intranasal pathway, these drugs or particles can be absorbed through mucous membrane inside nasal olfactory region, and then delivered into the brain directly through the cribriform plate. However, what cannot be overlooked is the short resident time of biologics in the nasal cavity for absorption, which decreases the concentration of drug in brain and compromises the therapeutic effect for diseases like Parkinson’s disease [25, 26]. With the development of nanomedicine, nanoparticles have emerged as a promising approach for delivering therapeutic agent across the BBB [27–30]. Lipid nanoparticles with solid matrix possess some advantages over other colloidal systems such as liposomes, emulsion, micro/nanoparticles, which include possibility of controlled release, drug targeting, increased stability, high payload, incorporation of both hydrophilic and hydrophobic drugs, and low biotoxicity [31, 32]. The gelatin nanoparticle used in this study is a type of gelatin-cored nanostructured lipid carrier with high stability, strong penetration, encapsulating efficiency, loading capacity as well as bioactivity [33]. It is reported that gelatin nanoparticles are excellent carriers for targeted delivery, making it possible to delivery therapeutics to special tissue effectively without compromising drug stability and concentration [34, 35].

The current study investigated whether SP-GNP can be delivered into CNS to play a neuroprotective role on hemiparkinsonian rats by inhibiting activation of JNK pathway and decrease dopaminergic neuron apoptosis. In this study, physicochemical properties of the prepared gelatin nanoparticles, including micromorphology, particle size, Zeta potential, encapsulating efficiency and loading capacity, were characterized. The growth promoting effect of SP solution and SP-GNP on 6-OHDA diseased PC-12 cells were assessed by MTT assays and flow cytometry analysis. The neuroprotective effect and the inhibition of JNK pathway activation and dopaminergic neuron apoptosis by intranasally administrated SP-GNP on hemiparkinsonian rats were evaluated by behavioral assessment, immumohistochemical staining and Western Blot analysis.

## Materials and Methods

### Materials and animals

Consent and approval for this investigation were obtained from the Laboratory Animal Ethics Committee of Wenzhou Medical University & Laboratory Animal Centre of Wenzhou Medical University (Wenzhou, Zhejiang, China). Rats were provided by the Laboratory Animal Centre of Wenzhou Medical University. Male SD rats weighed around 300~320g were used in the study. Two to three animals were housed per stainless steel cage on a 12-h light/12-h dark cycle in an air-conditioned room at 22°C, and check daily by the animal care staff. A standard commercial rat chow and water were available *ad libitum*.

### Preparation and characterization of SP-GNP and blank GNP

**Fig 1:**
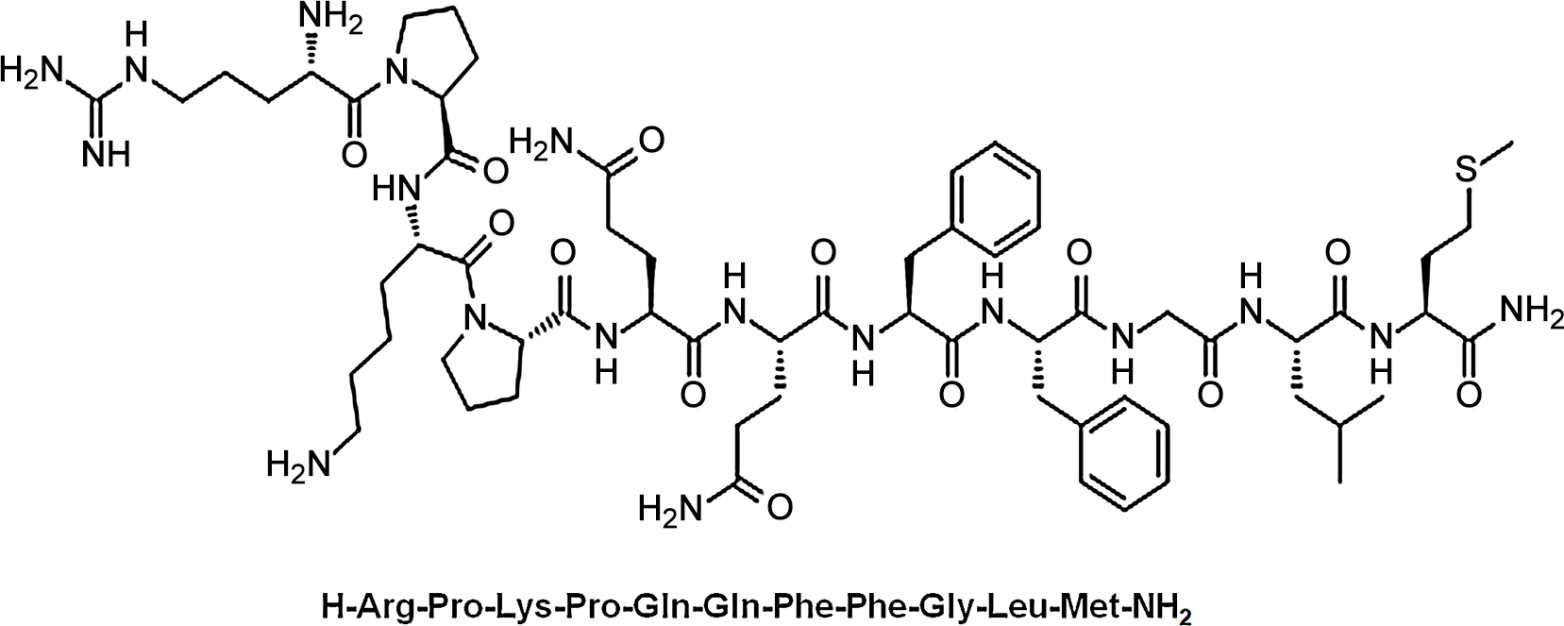
Molecular structure of SP.

All reagents used in this study were commercially available. SP was purchased from GL Biochem (Shanghai) Ltd. Its molecular structure is shown in Fig 1 and its molecule weight was 1347. The SP-GNP and blank GNP were prepared using water-in-water emulsion and freeze-drying technique [36, 37]. Briefly, high concentration SP was dissolved in 1 ml of 20% w/v Poloxamer 188–grafted heparin copolymer solution. The solution was added into 2 mL of 2.0% w/v gelatin solution to produce a homogeneous mixture. Under sonication (110 w, 15°C) using a probe sonicator, D, L-glyceraldehyde was injected into the mixture until its final concentration reached 0.1% w/v to initiate the cross-linking reaction. The mixture solution was bathed at 5°C under magnetic stirring at 2500 rpm for 5 h to form the SP gelatin nanoparticles suspension. The suspension was lyophilized to gain SP gelatin polymeric nanoparticles (SP-GNs) powder. Then lyophilized SP-GNs powder was dispersed in solution containing HSPC, trehalose and cholesterol. By sonication (90 w, 20 s) at 25°C, the mixture suspension was then lyophilized to gain lyophilized powder containing SP-GNP, which was reconstituted in double-distilled water to form SP-GNP suspension for administration.

Blank GNP (using gelatin solution instead of SP gelatin solution in preparation) and SP solution (SP dissolved in 0.9% NaCl solution) were also prepared for the following experiments. The final SP concentration in SP-containing solution was 2 mg/mL.

The morphologies of loaded or unloaded gelatin polymeric nanoparticles and gelatin nanoparticles were determined using a scanning electron microscopy (SEM) (X-650, Hitachi Co. Ltd., Tokyo, Japan). Their size and zeta potential values were determined by dynamic light scattering using a Zeta Potential/Particle Sizer Nicomp™ 380 ZLS (PSS. Nicomp, Santa Barbara, CA, USA).

To determine the encapsulating efficiency of SP-GNs and SP-GNP, approximately 1.5 mL of the SP-GNs or SP-GNP dispersion was placed in microtubes and was centrifuged at 10,000 g for 40 min. The supernatant was then collected and diluted for content determination of SP using an ELISA kit. The drug encapsulation efficiency was calculated as indicated below. The analyses were performed in triplicate.

Encapsulation efficiency (%) = (total amount of drug − amount of drug in supernatant) / total amount of drugs added initially × 100%

### Experiments *in vitro*

#### Cell culture

Male rat adrenal pheochromocytoma PC-12 cell was used for the *in vitro* studies. PC-12 cell was cultured in DMEM high-glucose medium with 10% FBS and 1% penicillin as well as streptomycin in humidified incubator (Thermo) containing 5% CO_2_ at 37°C. The cells in their logarithmic growth phase were harvested with trypsin for further experiments.

#### MTT assay

The growth enhancement of SP on 6-OHDA diseased PC-12 cells was confirmed by MTT assay. First of all, the suitable concentration of 6-OHDA to induce the disease model was determined. All cells were cultured in 96 well plates for 24 hours with a density of 5000 cells per well. With blank PC-12 cells as the control, four levels of 6-OHDA concentrations (25, 50, 75, 100 μmol/L) were incubated with PC-12 cells for 24 hours before 10μL of 5mg/mL of MTT [3-(4,5-dimethylthiazol-2-yl)-2,5-diphenyl-tetrazolium bromide] was added into each well and incubated for 4 hours. Then 100 μL of Formanzan solution was added into each well and incubated for another 4 hours to dissolve the crystals occurred in each well. Next, the plate was put in a microplate reader to measure the optical density at 526 nm and quantify the extent of cell viability. The lower extent of cell viability, the more damage 6-OHDA was done to the cells. The concentration causing the lowest extent of cell viability was chosen to set up the diseased cell model in the following assays.

Next was to choose the suitable SP concentration that could enhance cell growth. All cells were cultured in 96 well plates for 24 hours with a density of 5000 cells per well. With blank PC-12 cells as the control, six levels of SP concentrations (0. 1, 1, 10, 100, 1000, 10000 nmol/L) were incubated with PC-12 cells for 24 hours before 10μL of 5mg/mL of MTT [3-(4,5-dimethylthiazol-2-yl)-2,5-diphenyl-tetrazolium bromide] was added into each well and incubated for 4 hours. Then 100 μL of Formanzan solution was added into each well and incubated for another 4 hours, dissolving the crystals occurred in each well. Next, the plate was put in a microplate reader to measure the optical density at 526 nm and quantify the extent of cell viability. The higher extent of cell viability, the better effect SP was on cells. The concentrations raised the extent of cell viability were proved to enhance cell growth and were used in the following assays.

The final step was to investigate if SP solution and SP-GNP could enhance growth on diseased cells. All cells were cultured in 96 well plates for 24 hours with a density of 5000 cells per well. With blank PC-12 cells as the control, 6-OHDA in the most suitable concentration was used on cells for 24 hours to induce the diseased cell model. Then, different concentrations of SP solution and SP-GNP were incubated with PC-12 cells for 24 hours before 10μL of 5mg/mL of MTT [3-(4,5-dimethylthiazol-2-yl)-2,5-diphenyl-tetrazolium bromide] was added into each well and incubated for 4 hours. Then 100 μL of Formanzan solution was added into each well and incubated for another 4 hours, dissolving the crystals occurred in each well. Next, the plate was put in a microplate reader to measure the optical density at 526 nm and quantify the extent of cell viability. The higher extent of cell viability, the better effect SP solution or SP-GNP was on cells.

#### Flow cytometry

All cells were cultured in 12 well plates for 24 hours with a density of 50000 cells per well. With blank PC-12 cells as the control, 6-OHDA was added to cells for 24 hours to induce the diseased cell model. Different concentrations of SP solution and SP-GNP were then incubated with PC-12 cells for 24 hours before the cells were resuspended with 195μL binding buffer. Next, 5 μL of Annexin V-FITC was added and incubated for 10 minutes at room temperature in the dark. Then, the cells were resuspended with 190μL binding buffer again into tubes and 10 μL of Propidium Iodide (PI) was added. Finally, the tubes were placed in ice and the extent of cell apoptosis was detected in the Annexin V-FITCL and PI channels of the flow cytometer. Apoptotic cells were stained red with Annexin V-FITC, and necrotic cells were stained green with PI, while normal cells were not stained. Lower extent of cell apoptosis suggests better enhancement of SP solution or SP-GNP on cells.

### Experiments *in vivo*

#### Hemiparkinsonian rat model

**Fig 2:**
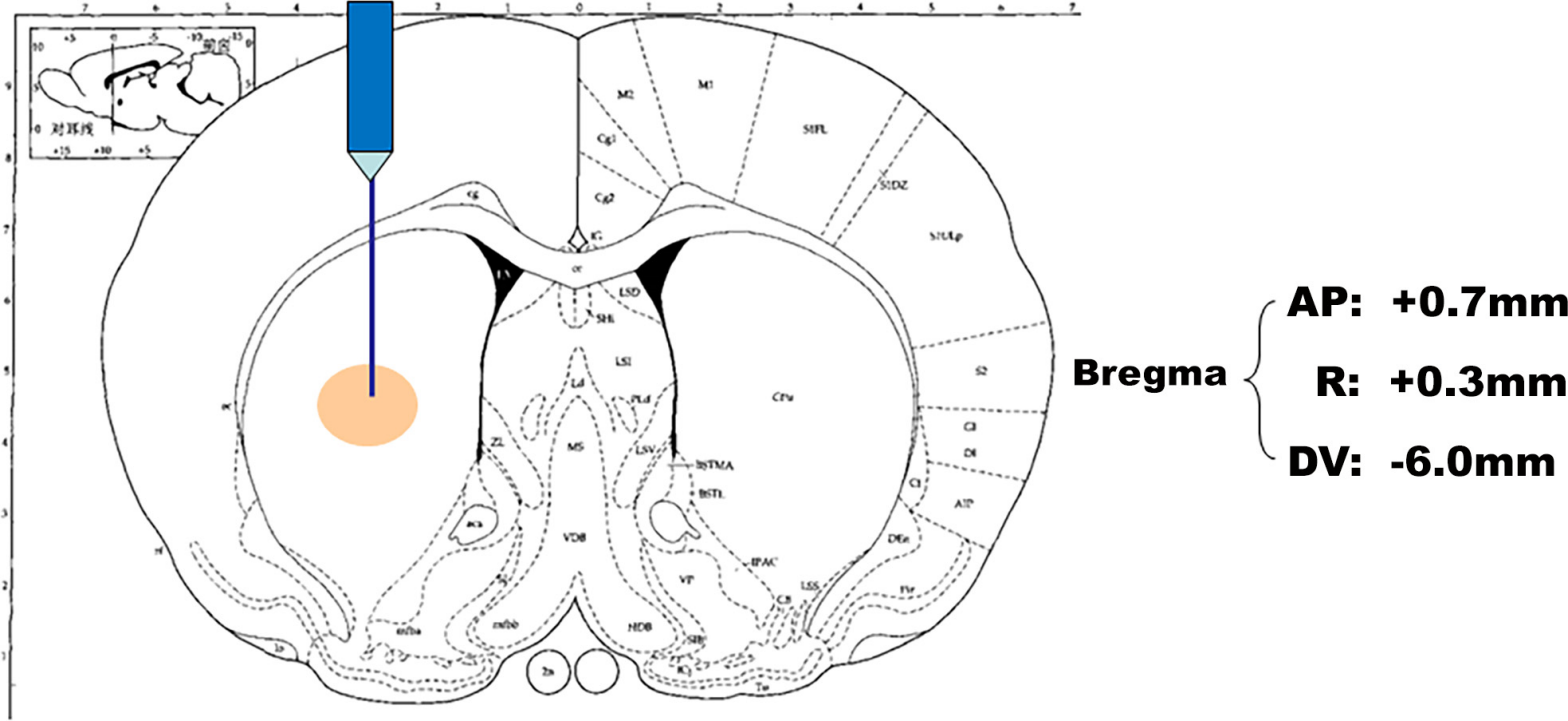
Creation of hemiparkinsonian rat model. The anesthetized SD rats were placed on stereotaxic apparatus and then injected with 12 μL 6-OHDA solution (or vehicle for sham animals) in the right-side striatum. AP: Distance after the fontanelle; R: Movement to the right side; DV: Depth value.

As seen in Fig 2, SD rats were anesthetized with pentobarbital sodium (60 mg/kg) and then injected with 12 μL 6-OHDA solution in the right-side striatum (or vehicle for sham animals) by use of the stereotaxic apparatus [38, 39]. Gentamicin was given after those procedures to prevent possible infection.

Four weeks after 6-OHDA solution injection, the behavior evaluation of apomorphine-induced rotations was conducted to determine if the hemiparkinsonian rat model was successfully set up. The rats were injected with apomorphine (0. 5 mg/kg) subcutaneously, and then both contra- and ipsi-lateral full-body rotations in the following 30 minutes were recorded. The rats had over 7 full-body contralateral rotations per minute were considered as successful hemiparkinsonian model rats (PD rats), which were used in the following experiments.

#### Treatment of hemiparkinsonian rats

After the behavior evaluation, sham SD rats and PD rats were divided into six groups randomly (n = 6/group) and started daily treatment for two weeks. As seen in Table 1, group 1 was sham rats with intranasal PBS treatment. Group 2–4 were PD rats which received intranasal PBS, blank GNP and SP solution respectively. Group 5 and group 6 were PD rats received SP–GNP intranasally and intravenously.

**Table 1:** Groups designed for two-week daily treatment (n = 6/group).

Group	Rats	Administration
1	Sham	*in*.PBS
2	Hemiparkinsonian	*in*.PBS
3	Hemiparkinsonian	*in*.Blank GNP
4	Hemiparkinsonian	*in*.SP solution
5	Hemiparkinsonian	*iv*.SP-GNP
6	Hemiparkinsonian	*in*.SP-GNP

*Note*: *in*. represents intranasal administration; *iv*. represents intravenous administration.

After the two-week daily treatment, the experimental rats were injected subcutaneously with apomorphine (0.5 mg/kg) to evaluate their neuro-recovery extent. During the 30 minutes after apomorphine injection, rats’ all contra- and ipsi-lateral full-body rotations were measured. The rat groups with low rotations suggested some neuro-recovery.

The rats were then all sacrificed and their brains were collected for coronal sectioning across the striatum region as well as the SNc region. Half of them were embedded in paraffin and sectioned for histopathological study and immunohistochemical staining, while the rest were dissected and homogenized in cold lysis buffer for Western blot assessment.

#### Histopathological study

The paraffin sections were handled with conventional deparafinization and hydration steps before incubating in hematoxylin for 7 min. Next, the sections were washed with cold running water for 9 min and soaked in 95% ethanol for 5 sec, and incubated in eosin for 30 sec. Then a drop of permount and a glass slip were added to the sections. Finally, the sections were placed under optical microscope to study the microstructure.

#### Immunohistochemical staining

The immunohistochemical staining with anti-TH antibody, anti-p-c-Jun antibody and anti-Caspase-3 antibody was conducted to evaluate therapeutic effect of different SP preparations on hemiparkinsonian rats.

Tyrosine hydroxylase (TH) is the rate-limiting enzyme to catalyze the hydroxylation of L-tyrosine to L-3,4-dihydroxyphenylalanine (L-DOPA), the precursor to dopamine, norepinephrine and epinephrine. TH is considered a marker for dopaminergic neurons [40]. Higher ratio of TH staining suggests less loss of dopaminergic neurons. Phosphorylated c-Jun protein (p-c-Jun) is a vital protein in JNK pathway. As the primary endpoint shear enzyme in the process of cell apoptosis, Caspase-3 plays an irreplaceable part during cell apoptosis. The ratio of p-c-Jun and Caspase-3 staining suggests their levels in SN regions.

#### Western blot assessment

Western blot assessment was carried out to evaluate the TH, p-c-Jun and Caspase-3 levels in SN area of experimental rats. Proteins were extracted in an ice bath and their levels were assessed using standard biochemical procedures [4]. The band densities were quantified by densitometry (Quantity One software, Bio-Rad, Hercules, CA, USA).

### Statistical analysis

Statistically significant difference for multiple groups was determined using a one way ANOVA with a Newman-Keuls post-hoc test. Statistical significance between individual groups was determined using a Mann-Whitney U test. All testing was done with Statistical Program for Social Sciences (Spass) 19. Difference was considered statistically significant when the p-value was less than 0. 05.

## Results

### Physicochemical properties and bioactivity of gelatin nanoparticle

**Fig 3:**
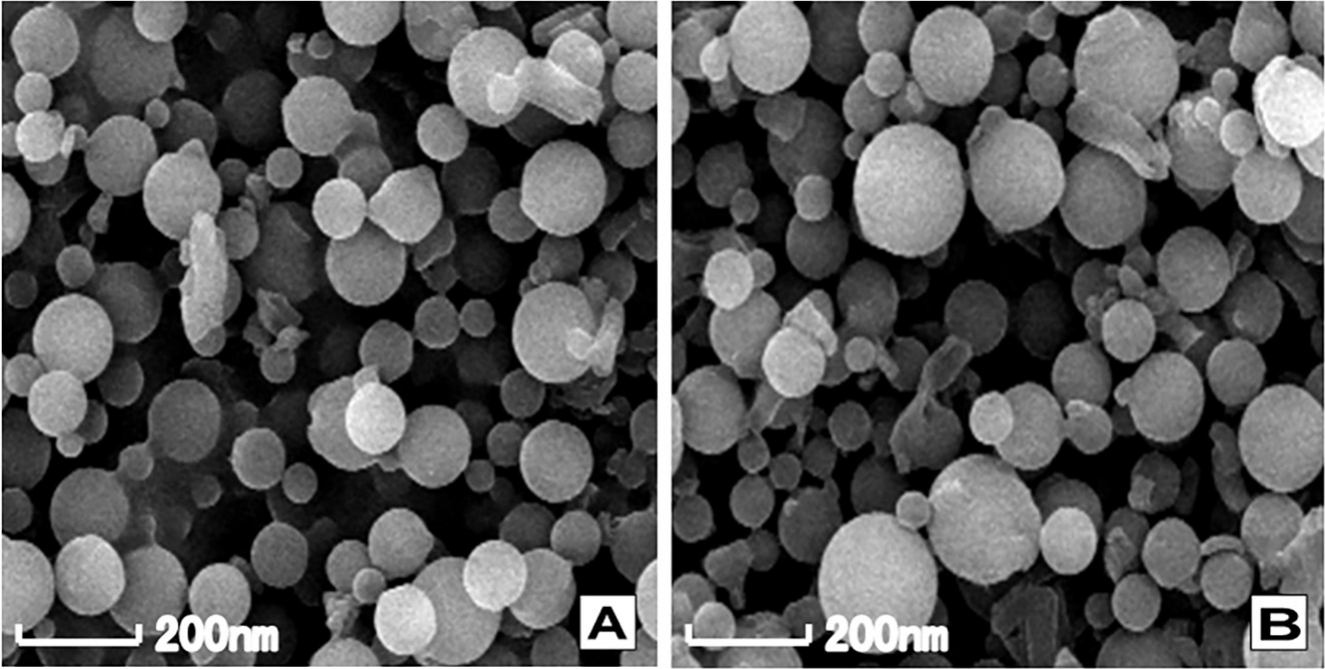
Scanning electron micrographs of blank GNP (A) and SP-GNP (B). SEM micrographs showed both blank GNP and SP-GNP were uniform in shape, and the dynamic light scattering demonstrated that both blank GNP and SP-GNP were uniform in size.

From SEM micrographs, the blank GNP and SP-GNP were uniform in shape and size (Fig 3). Characterizations of GNs and GNPs loaded with or without SP were shown in Table 2. The dynamic light scattering results demonstrated that the average particle size of blank GNs and GNPs were 87±1.01 nm and 136±1.32 nm respectively. The polydispersibility index (PDI) represents the distribution of particle size. Low PDI values were observed for both of blank GNP and SP-GNP (Table 2), indicating blank GNP and SP-GNP approached a monodisperse stable system. After SP loading, the mean diameters of nanoparticle increased, not exceeding 200 nm (Table 2). The zeta potential is an important index evaluating the physical stability of nanoparticles. Nanoparticles with high absolute value of zeta potential are electrically stable while those with low absolute value of zeta potential tend to be less stable. As shown in Table 2, both of the blank GNP and SP-GNP possessed stronger negative charge on the surface than blank GNs and SP-GNs, indicating the stabilization of phospholipid-coated nanoparticles. Furthermore, SP-GNP showed better encapsulation efficiency and loading capacity than SP-GNs (Table 2).

**Table 2:** Characterization of GNs and Lip loaded with or without SP (n = 6).

Formulation	Particle size (nm)	PDI	Zeta Potential (mV)	Encapsulation Efficiency (%)	Load Capacity (%)
Blank GNs	107±1.01	0.221±0.034	-20.3±0.8	-	-
Blank GNP	166±1.32^*^	0.145±0.021^*^	-37.6±1.4^*^	-	-
SP-GNs	121±1.21	0.234±0.041	-18. 2±0.5	35.8±1.6	1.34±0.05
SP-GNP	172±1.52^#^	0.107±0.013^#^	-29.6±1.2^#^	93.3±1.4^#^	5.2±0.02^#^

*Note*: PDI, Polydispersity Index.

^*^represents P<0. 05 *vs* blank GNs,

^#^represents p<0. 05 *vs* SP-GNs.

### MTT result

**Fig 4:**
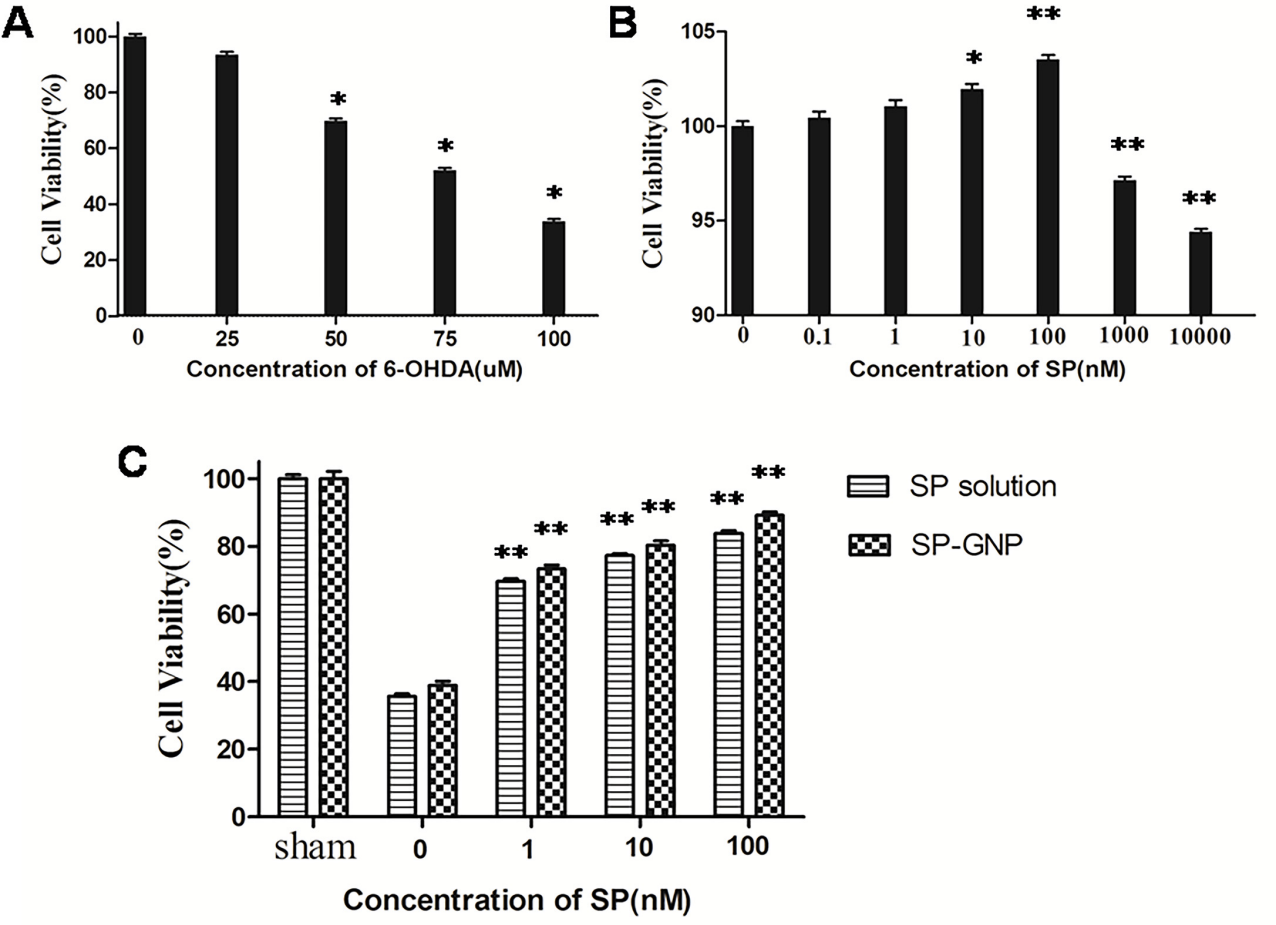
Results of MTT assay on PC-12 cells. (A) Growth inhibition of 6-OHDA in different concentrations on PC-12 cells. * represents p <0.05 *vs* sham cells. (B) Growth enhancement of SP solution in different concentrations on PC-12 cells. * represents p <0.05 *vs* sham cells. ** represents p <0.01 *vs* sham cells. (C) Growth enhancement of SP solution and SP-GNP in different concentrations on 6-OHDA diseased PC-12 cells.** represents p<0.01 *vs* 6-OHDA diseased cells without SP treatment.

The growth inhibition of 6-OHDA in different concentrations on PC-12 cells was shown in Fig 4A. The cell viability decreased with the increased concentration of 6-OHDA, indicating the role of 6-OHDA in cell apoptosis. Cells treated with 6-OHDA concentration of 100μmol/L showed significantly lower cell viability (p<0. 05) than the sham cells.

Fig 4B showed the growth enhancement of SP solution in different concentrations on PC-12 cells. With the concentration of 6-OHDA increasing, the cell viability showed increasing trend when SP concentration was lower than 100nM, but showed a declined trend when SP concentration was up to 1μM or 10μM. From the result, SP can enhance cell growth in low dose while inhibit cell growth in high dose.

Fig 4C revealed the growth enhancement of SP solution and SP-GNP on 6-OHDA treated PC-12 cells. These PC-12 cells treated with SP solution or SP-GNP treatment demonstrated higher cell viability (p<0. 05) than those without SP treatment. This illustrated that SP of certain concentration could reduce cell apoptosis caused by 6-OHDA and enhance cell growth. Furthermore, the cell viability with SP-GNP treatment was much higher than those with SP solution treatment (p<0.01). Compared with SP solution, SP-GNP can play a better role to mediate SP entering the cells and execute its role in the inhibition of apoptosis, showing a better therapeutic effect on 6-OHDA treated cells.

### Flow cytometry

**Fig 5:**
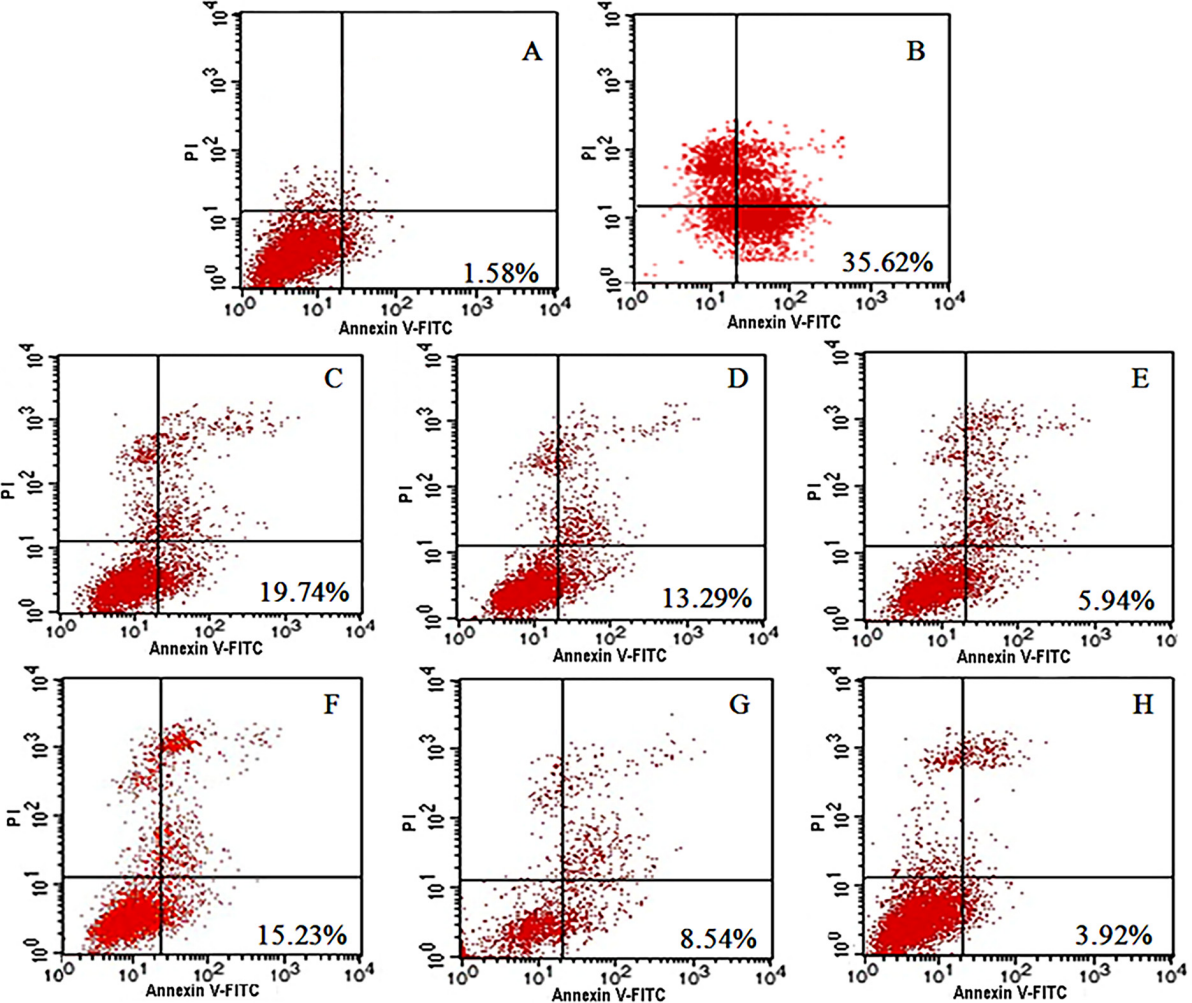
Flow cytometry results on PC-12 cells. (A) Blank PC-12 cells. (B) 6-OHDA diseased PC-12 cells without treatment. (C) 6-OHDA diseased PC-12 cells with 1nM SP solution treatment. (D) 6-OHDA diseased PC-12 cells with 10nM SP solution treatment. (E) 6-OHDA diseased PC-12 cells with 100nM SP solution treatment. (F) 6-OHDA diseased PC-12 cells with 1nM SP-GNP treatment. (G) 6-OHDA diseased PC-12 cells with 10nM SP-GNP treatment. (H) 6-OHDA diseased PC-12 cells with 100nM SP-GNP treatment.

Fig 5 revealed the ratio of apoptosis cells and growth enhancement of SP administrated groups on 6-OHDA treated PC-12 cells. With SP treatment, the ratio of apoptosis in 6-OHDA treated cells decreased, compared with those without SP treatment. Furthermore, the 6-OHDA treated cells with SP-GNP achieved lower ratio of apoptosis than those with SP solution treatment. Compared with SP solution, SP-GNP showed better enhancement in SP penetration into the cells, and exhibited stronger inhibition on cell apoptosis.

### Behavioral evaluation of PD rats after two-week treatment

The apomorphine-induced rotations following two-week daily treatment of SP in each experimental group were consistent with the DA levels in the diseased brain. As seen in Table 3, the order of the reduction in apomorphine-induced rotations was: intranasal SP-GNP > intravenous SP-GNP > intranasal SP solution > intranasal Blank GNP > intranasal PBS ≈ sham group. The hemiparkinsonian rats received intranasal blank GNP treatment showed little difference from those with intranasal PBS treatment. However, the rotations of the hemiparkinsonian rats with SP administration were lower than those without SP administration, demonstrating that SP could enhance the recovery of diseased dopaminergic neurons in hemiparkinsonian rats. In the meantime, hemiparkinsonian rats treated with intranasal SP-GNP (p = 0.001) and intravenous SP-GNP (p = 0.02) showed a significant decrease on apomorphine-induced rotation than hemiparkinsonian rats with intranasal PBS solution treatment, while those with intranasal SP solution and blank GNP treatment showed a trend of improvement without statistical significance (p = 0.15 and p = 0.35 respectively). From the result, SP-GNP could play a better role on the recovery of diseased dopaminergic neurons in hemiparkinsonian rats than intravenous SP-GNP and intranasal SP solution.

**Table 3:** Apomorphine-induced rotations after two-week daily SP treatment (n = 6/group).

Group	Rats	Administration	Rotations (r/min)
1	Sham	*in*.PBS	0
2	Hemiparkinsonian	*in*.PBS	7. 8±0. 6
3	Hemiparkinsonian	*in*.Blank GNP	7. 4±0. 8
4	Hemiparkinsonian	*in*.SP solution	6. 8±1. 5
5	Hemiparkinsonian	*iv*.SP-GNP	6. 2±1. 3*
6	Hemiparkinsonian	*in*.SP-GNP	4. 7±1. 9**

*Note*: *in*. represents intranasal administration; *iv*. represents intravenous administration.

* represents p<0.05 *vs* hemiparkinsonian group;

** represents p<0.01 *vs* hemiparkinsonian group.

### Histopathological observation

**Fig 6:**
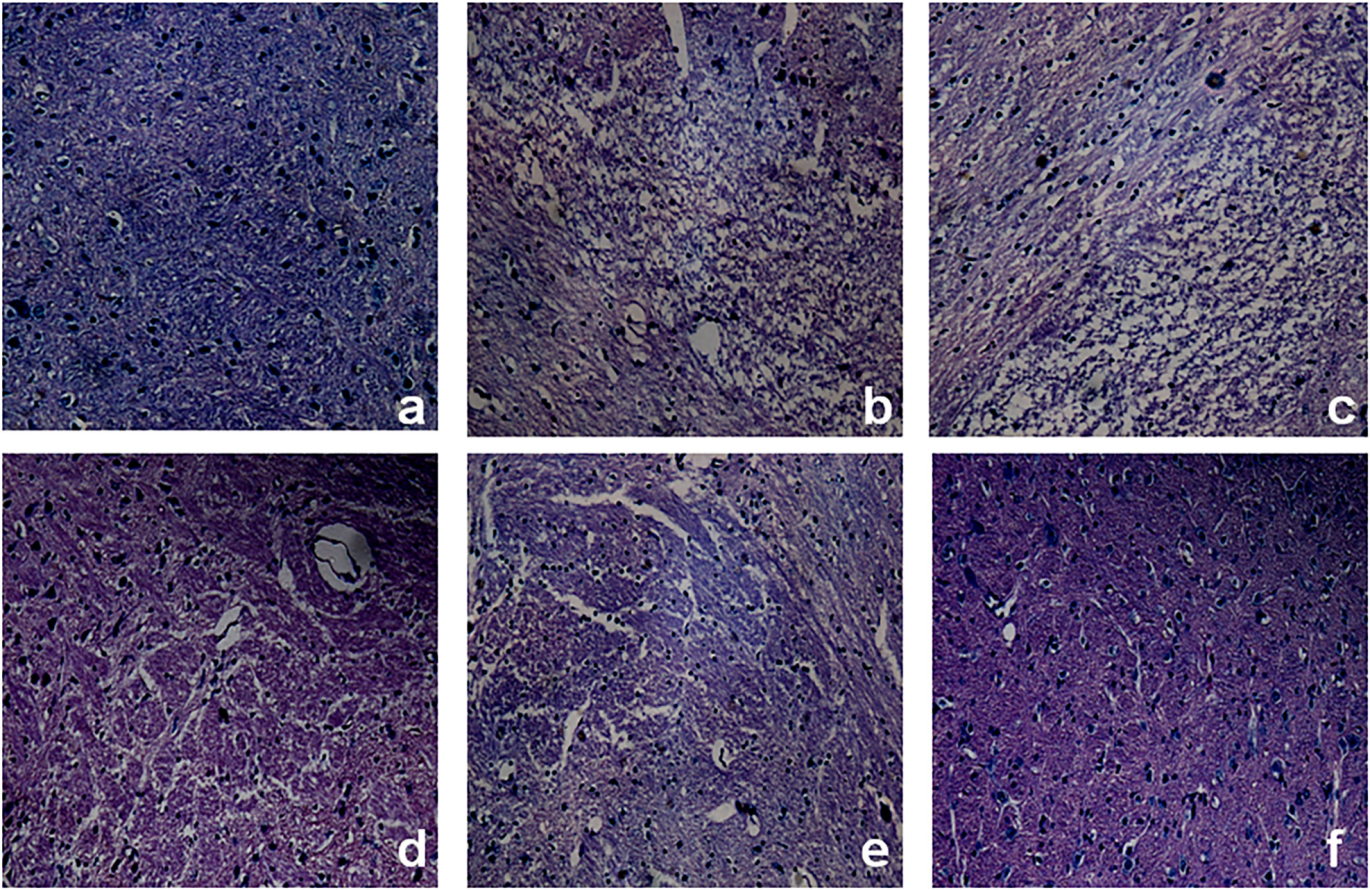
Histopathological HE staining. (a) Sham SD rats. (b) Hemiparkinsonian rats with intranasal PBS treatment. (c) Hemiparkinsonian rats with intranasal blank GNP treatment. (d) Hemiparkinsonian rats with intranasal SP solution treatment. (e) Hemiparkinsonian rats with intravenous SP-GNP treatment. (f) Hemiparkinsonian rats with intranasal SP-GNP treatment.

The HE staining of the right substantia nigra in each group of rats was shown in Fig 6. Compared with normal group (Fig 6A), hemiparkinsonian model group and the group receiving blank GNP group (Fig 6B and 6C) showed apparently reduced cell numbers, with large number of glial cell proliferation, inflammatory cell infiltration and connective tissue proliferation observed. After SP treatment, there was an obvious increase of neuron number and a reduction in glial cell proliferation, inflammatory cell infiltration and connective tissue hyperplasia. Compared with the rats with SP solution treatment and intravenous SP-GNP treatment (Fig 6D and 6E), rats with intranasal SP-GNP treatment (Fig 6F) possessed a larger number of neurons with regular arrangement. The glial cell proliferation, inflammatory cell infiltration and connective tissue proliferation were obviously limited in intranasal SP-GNP group, following with intravenous SP-GNP and intranasal SP solution groups. Result from the histopathological observation further supported the data of behavioral evaluation mentioned in Table 3.

### Immunohistochemical staining of TH, p-c-Jun and Caspase-3 levels in diseased SN region

**Fig 7:**
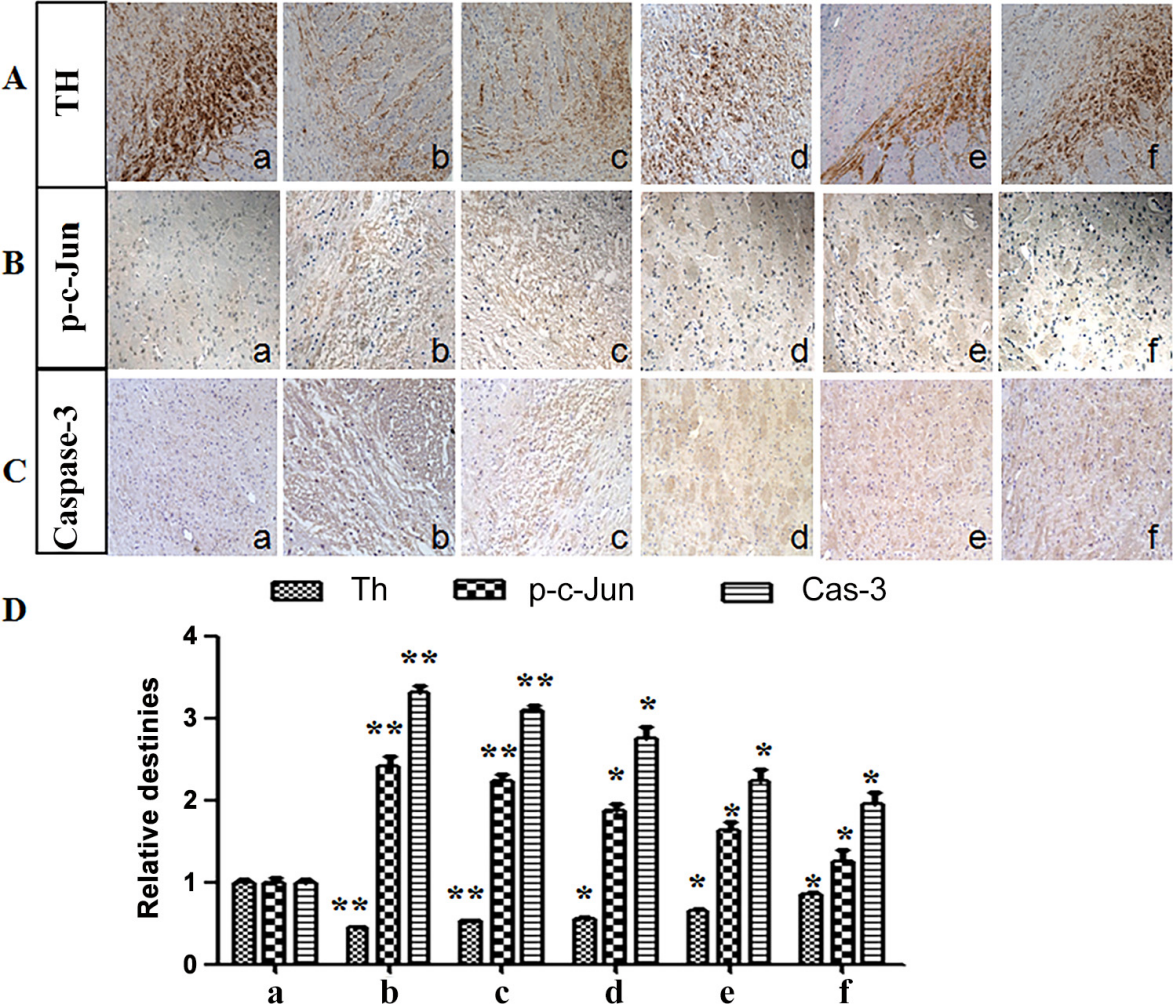
Immunohistochemical staining. (a) Sham SD rats with intranasal PBS treatment. (b)PD rats with intranasal PBS treatment. (c)PD rats with intranasal blank GNP treatment. (d) PD rats with intranasal SP solution treatment. (e) PD rats with intravenous SP-GNP treatment. (f) PD rats with intranasal SP-GNP treatment. * represents p<0.05 *vs* hemiparkinsonian group (Group b), ** represents p<0.

TH immunohistochemical staining was shown in Fig 7A. Image-Pro Plus 6. 0 was used to quantify the cells, the staining area and the staining degrees. TH immunohistochemistry in diseased SN region revealed severe loss of dopaminergic cells in hemiparkinsonian group and in the blank GNP treated group, compared with sham group. More TH-positive staining was seen in SP administration groups (p<0. 05) than in hemiparkinsonian group, illustrating the enhancement of SP on the recovery of diseased dopaminergic neurons. Among SP administrated groups, intranasal SP-GNP group (p<0. 01) showed more TH-positive staining than intranasal SP solution group and intravenous SP-GNP group. From the result, intranasal SP-GNP can effectively mediate SP into the brain through the nose and exhibit its neuro-recovery function in the damaged areas of the brain.

From p-c-Jun immunohistochemistry in diseased SN region (Fig 7B), sham-PBS/IN group showed little staining, while hemiparkinsonian group and the blank GNP group showed extremely high levels of staining. It indicated that p-c-Jun was rarely expressed in normal circumstances but expressed in large amount when PD occurred. Less staining was seen in SP administration groups than hemiparkinsonian group (p<0.05), indicating that SP might block the JNK pathway activation, inhibit the expression of p-c-Jun, and thus slow down the PD disease process. Among SP administration groups, intranasal SP-GNP group showed less staining than intranasal SP solution group and intravenous SP-GNP group (p<0.01), demonstrating the effective inhibition of intranasal SP-GNP on the JNK pathway and the p-c-Jun expression in the brain.

As shown in Fig 7C, Caspase-3 immunohistochemistry in diseased SN region revealed little staining in sham-PBS/IN group and extremely high levels of staining in hemiparkinsonian group and the blank GNP group. From the result, Caspase-3 was rarely expressed in normal circumstances but expressed in large amount when PD occurred. Less staining was observed in SP administration groups than in hemiparkinsonian group (p<0.05). Therefore, SP could reduce neuron apoptosis, inhibit the expression Caspase-3, and promote the recovery of the diseased neurons. Among SP administration groups, intranasal SP-GNP group showed less staining than intranasal SP solution group and intravenous SP-GNP group, further supporting the efficiency SP-GNP for SP delivery into the diseased SN region.

### Western Blot assessment of TH, p-c-Jun and Caspase-3 levels in diseased SN region

**Fig 8:**
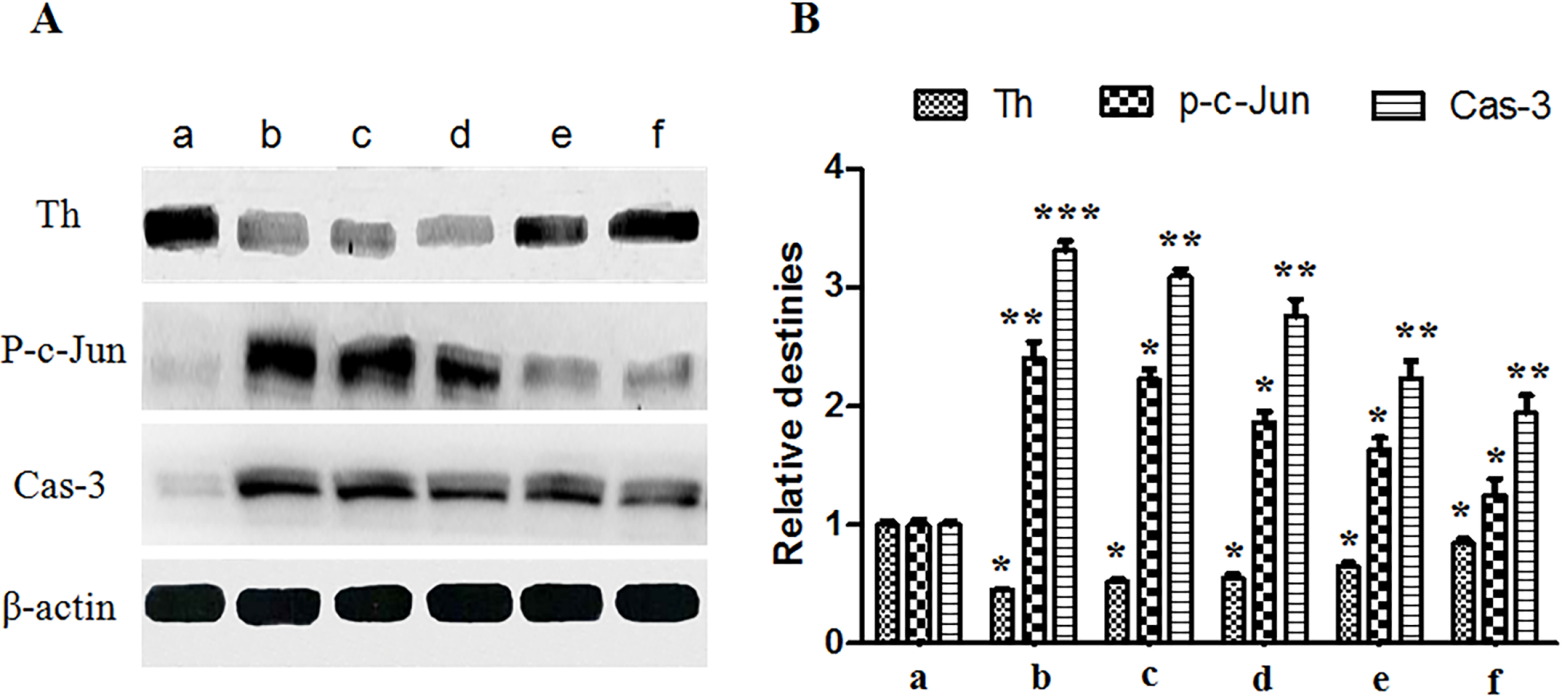
Western blot assessment. PD rats with intranasal PBS treatment. (c)PD rats with intranasal blank GNP treatment. (d) PD rats with intranasal SP solution treatment. (e) PD rats with intravenous SP-GNP treatment. (f) PD rats with intranasal SP-GNP treatment. * represents p<0.05 *vs* hemiparkinsonian group (Group b), **01*vs* hemiparkinsonian group (Group b).

Western Blot was conducted to analyze the TH, p-c-Jun and Caspase-3 levels in diseased SN region quantitatively. As shown in Fig 8, TH levels in hemiparkinsonian group and the blank GNP group were much less than those in the sham group. Compared with hemiparkinsonian group, SP treatment groups showed increased TH levels (p<0.05), proving the SP therapeutic action for diseased dopaminergic neurons. Among SP administration groups, intranasal SP-GNP group showed higher TH level than the intranasal SP solution group and intravenous SP-GNP group, suggesting the effective neuro-recovery function of intranasal SP-GNP in the brain.

As for the p-c-Jun and Caspase-3 levels in diseased SN region, the sham group showed both very low concentration of p-c-Jun and Caspase-3 while hemiparkinsonian group and the blank GNP group revealed quite high level of them. From the result, p-c-Jun and Caspase-3 were rarely expressed in normal circumstances but expressed in large amount when PD developed. Compared with hemiparkinsonian group, SP treated groups showed decreased levels in p-c-Jun and Caspase-3 (p<0.05). It suggested that SP could play a role in reducing neuron apoptosis and inhibiting the expression of p-c-Jun and Caspase-3. Among SP treated groups, the p-c-Jun and Caspase-3 levels in intranasal SP-GNP group were much lower than intranasal SP solution group and intravenous SP-GNP group, demonstrating the enhancement of the intranasal SP-GNP for SP penetration into the brain. In the meantime, the relative destiny of p-c-Jun level was lower than that of Caspase-3 level in hemiparkinsonian group and other groups with treatment. All the results from Western Blot and immunohistochemical staining were consistent with the results of histopathological observation and behavioral evaluation of PD rats.

## Discussion

Current therapies for Parkinson’s disease have limitations due to the presence of BBB which prevents therapeutic chemicals or biologics entering the brain. As a member of the tachykinin peptide family, SP can play an important role in treating diseased dopaminergic neuron through deactivation of JNK pathway and inhibition of neuron apoptosis. But SP is a macromolecule which can not penetrate the BBB. In this study, a novel gelatin nanoparticle encapsulating SP was prepared to investigate the role of SP in enhancing neuro-recovery on hemiparkinsonian rats via the noninvasive intranasal route.

### Gelatin nanoparticle mediated intranasal delivery of therapeutics to CNS

Therapeutic biologic particles with a size below 300 nm can bypass BBB and gain access to the brain directly through the cribriform plate, and then execute their therapeutic effects [26]. They are believed to gain access to the CNS after intranasal administration via the following pathways: the systemic circulation in which the drug has to cross the BBB; the olfactory pathway in which the drug is taken up by the olfactory epithelium and enter the olfactory bulb; and the trigeminal pathway in which the drug is transported via the trigeminal nerve system [41, 42]. However, their residence time at the delivery site is quite limited due to the mucociliary clearance mechanisms, which restricts their concentration level in CNS and their biological activity as well.

Researchers have demonstrated that lipid nanoparticles mediated intranasal delivery of therapeutics can gain access directly into the brain and achieve effective treatment on brain diseases such as cerebral ischemic [43] and Parkinson’s disease [44]. It has been reported that nonionic surfactants, like Poloxamer 188, might increase transcellular transport of olanzapine by reduction of the barrier function of the mucous layer due to their ability to reduce the mucous viscosity and elasticity, or to modulate the tight junction [45].] In recent years, gelatin is adopted for nanoparticle preparation due to its biocompatibility, biodegradability, low immunogenicity and surface modification. Studies have shown that modified gelatin mixed with Poloxamer 188 can generate nanoparticles that may be used for intranasal drug delivery [46]. Intranasal administrated gelatin nanoparticle, which is prepared with Poloxamer 188 and gelatin, can bypass BBB and find their way right into CNS through the olfactory pathway and the trigeminal pathway, and then the loaded drugs can play therapeutic role in CNS. In the meantime, gelatin nanoparticle possesses stronger negative charge, which can reduce mucociliary clearance, extend the resident time at the delivery site and enhance the therapeutic effect when intranasally administrated [33–35].

### The role of SP in treating diseased dopaminergic neuron through deactivation of JNK pathway and inhibition of neuron apoptosis

From previous studies, SP administrated intracerebroventricularly in a suitable concentration can inhibit both of the activation of JNK pathway and the expression of caspase-3 [1, 16], showing significant functional improvement of Parkinson’s disease [9–11]. However, its therapeutic effect *in vivo* is limited due to the poor permeability into BBB when administered intravenously.

PC—12 cell, a monoamine cell derived from pheochromocytoma of the male rat adrenal medulla which expresses Tyrosine hydroxylase (TH) and synthesise dopamine inside of cell, is widely used to study Parkinson’s disease model *in vitro* [47]. In this study, PC-12 cells were used to examine the growth enhancement of SP *in vitro*. From the result, SP within a certain range of concentrations can enhance cell growth and decrease apoptosis, supporting the therapeutic application for Parkinson’s disease.

For *in vivo* experiment, 6-OHDA was injected into the right-side striatum of rats to create hemiparkinsonian rat model. Those rats were then divided into 6 groups and administrated SP solution or SP-loaded gelatin nanoparticle intranasally or intravenously. Finally, the rats were sacrificed and the levels of TH, p-c-Jun and caspase-3 were evaluated through immunohistochemical study and Western Blot analysis. From this study, SP can play an important role in deactivation of JNK pathway and inhibition of expression of capase-3, thus recovering the diseased dopaminergic neurons and decreasing the neuron apoptosis in hemiparkinsonian rats.

Though the levels of p-c-Jun and caspase-3 decrease when hemiparkinsonian rats are administrated with SP, it is still unknown whether the reduction of caspase-3 level is mainly caused by deactivation of JNK pathway or by the inhibition of expression of capase-3. Researchers showed that JNK signaling pathway downstream mitochondrial malfunction can lead to over-expression of Caspase-3 protein, ultimately leading to apoptosis or degenerative death of cells [13]. SP can regulate p38 MAPK pathway, JNK pathway, and activate ERK pathway, thereby regulating the expression of caspase family of proteins and cell apoptosis, and improving the symptoms of various brain diseases [15, 16]. It remains unclear whether deactivation of JNK pathway or inhibition of expression of capase-3 is the dominating factor in the role for decreasing the neuron apoptosis.

### Therapeutic effect of SP-GNP on 6-OHDA induced PC-12 cell and hemiparkinsonian rats

In this study, novel SP-GNP was prepared and administrated to 6-OHDA induced PC-12 cells and hemiparkinsonian rats via the noninvasive intranasal route. Then the therapeutic effects of SP-GNP and pure SP solution on 6-OHDA induced hemiparkinsonian rats were assessed by quantifying rotational behavior and the levels of tyrosine hydroxylase (TH), phosphorylated c-Jun protein (p-c-Jun) and Caspase-3 (Cas-3) expressed in substantia nigra (SN) region.

From the in vitro experiment, PC-12 cells under SP-GNP treatment showed better cell viability (Fig 4C) and lower degree of apoptosis (Fig 5) than those under SP solution treatment. Hemiparkinsonian rats under intranasal SP-GNP administration demonstrated better behavioral improvement (Table 3), neuron regeneration (Fig 6), higher TH levels in SN (Fig 7) along with much lower extent of p-c-Jun and Cas-3 (Fig 8) than those under intranasal SP solution administration. Compared with SP solution, SP-GNP showed more effective penetration for PC-12 cells and BBB, along with the stronger inhibition of cell apoptosis both *in vitro* and *in vivo*. Histopathological study also illustrated the good biocompatibility of SP-GNP with 6-OHDA treated neuron.

## Conclusions

To sum up, SP can enhance 6-OHDA induced dopaminergic neuron recovery in hemiparkinsonian rats through deactivation of JNK pathway and reduction of dopaminergic neuron apoptosis (S1 Fig). SP-GNP administrated intranasally may be an effective therapy choice for Parkinson’s disease.

## Supporting Information

S1 Fig Shows the mechanism diagram of using gelatin nanoparticle mediated intranasal delivery of neuropeptide substance P to enhance neuro-recovery in hemiparkinsonian rats. (TIF)

## Abbreviations

PD: Parkinson Disease
GNP: gelatin nanoparticles
SP: Substance P
SP-GNP: Substance P-loaded gelatin nanoparticles
MTT: 3-(4, 5-dimethylthiazol-2-yl)-2, 5-diphenyl-tetrazolium bromide
FCM: Flow cytometry
6-OHDA: 6-hydroxydopamine
TH: Tyrosine hydroxylase
p-c-Jun: Phosphorylated c-Jun protein
Cas-3: Caspase-3
SN: Substantia nigra
JNK: c-Jun N-terminalkinase
CNS: Central nervous system
DA: Dopamine
MAPK: Mitogen-activated protein kinase
BBB: Blood-brain barrier
GN: Gelatin polymeric nanoparticles
SP-GN: Substance P-loaded gelatin polymeric nanoparticles
L-DOPA: L-3, 4-dihydroxyphenylalanine
PDI: Polydispersibility index
