# Supplementary figures and images for: Using Gelatin Nanoparticle Mediated Intranasal Delivery of Neuropeptide Substance P to Enhance Neuro-Recovery in Hemiparkinsonian Rats
Source: PLoS One. 2016 Feb 19;11(2):e0148848. doi: 10.1371/journal.pone.0148848 (PMC4760767; doi:10.1371/journal.pone.0148848)

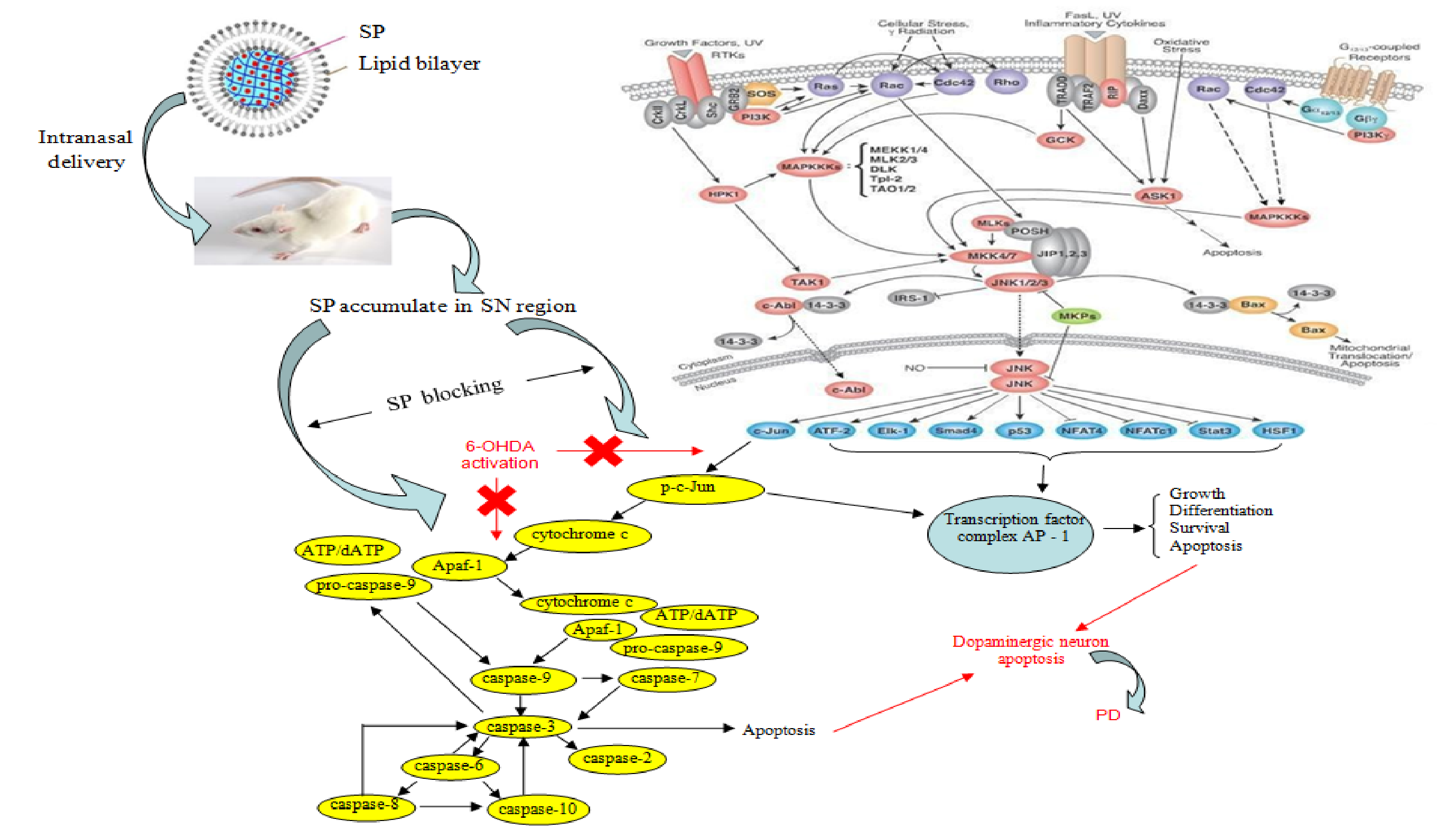

Supplement: S1 Fig — (TIF) [file pone.0148848.s001.tif]
